# First principles electronic transport study of triacetone triperoxide adsorbed on Ti_3_C_2_O_2_ and Ti_3_C_2_F_2_ MXene monolayers

**DOI:** 10.1186/s11671-026-04561-2

**Published:** 2026-04-21

**Authors:** Aleksandar Ž. Tomović, Ivana Djurišić, Miloš S. Dražić, Vladimir P. Jovanović, Radomir Zikic

**Affiliations:** University of Belgrade - Institute for Multidisciplinary Research, National Institute of the Republic of Serbia, Kneza Višeslava 1, Belgrade, 11030 Serbia

## Abstract

Triacetone triperoxide (TATP) is a highly volatile and extremely dangerous peroxide-based explosive synthesized from readily accessible precursor chemicals. Due to its non-nitro composition, TATP evades most conventional field-detection systems. In this work, we investigate the potential of Ti_3_C_2_O_2_ and Ti_3_C_2_F_2_ MXene monolayers as portable, low-power sensing platforms for TATP vapor detection using density functional theory (DFT) coupled with non-equilibrium Green’s function (NEGF) transport calculations. Three adsorption configurations (parallel and two orthogonal orientations) were systematically examined. TATP is adsorbed onto the surfaces of both MXenes via a multi-point hydrogen-bonding mechanism. This interaction yields net adsorption energies of −  0.45 eV and − 0.37 eV for Ti_3_C_2_O_2_ and Ti_3_C_2_F_2_, respectively. Density-of-states (DOS) and eigenstate analyses reveal HOMO-level pinning near the Fermi energy on Ti_3_C_2_O_2_, enabling detectable current modulation at low bias. Transport simulations yield currents on the order of 10 µA at 0.1 V, at which relative current changes are measurable and amount to 3.7% for Ti_3_C_2_F_2_ and 1% for Ti_3_C_2_O_2_. The involvement of TATP’s HOMO in the transport window for Ti_3_C_2_O_2_ at elevated bias provides an additional sensing mechanism. Our findings suggest that both Ti_3_C_2_O_2_ and Ti_3_C_2_F_2_ MXenes are promising building blocks for portable, resistive TATP detectors.

**Supplementary Information:**

The online version contains supplementary material available at 10.1186/s11671-026-04561-2.

## Introduction

Over the years, two-dimensional materials such as graphene, transition metal dichalcogenides, and MXenes have attracted attention as a platform for sensing toxic and dangerous gases owing to their excellent electronic properties [1–3]. The recent research has shown the ability of MXene-based resistive sensors to detect hazardous gases such as volatile organic compounds (VOCs), NH_3_, SF_6_, and SF_6_/N_2_ decomposition gases [4–9]. MXene sensors for explosives detection were based on the utilization of surface plasmon resonance [10], while their ability to act as a resistive sensor for explosives was not systematically explored yet.

The TATP, a dangerous and unstable explosive that readily evaporates, is produced using acetone and hydrogen peroxide [11]. TATP is an organic peroxide that contains no nitro groups, meaning it evades traditional detection systems designed for nitro-based explosives such as TNT and RDX. TATP’s very high vapor pressure at room temperature [12] and ease of synthesis have contributed to its widespread use in attacks over the past two decades (Paris, London, Surabaya, etc.). The TATP is usually detected using mass spectrometry [13], infrared [14] and Raman [14] spectroscopy, and microwaves [15]. While these methods provide accurate TATP detection, they lack portability and require sample collection, which incurs risk.

There is an ongoing effort to provide new TATP nanotechnology-based sensing methods that offer portability and ease of use [16–22]. Experimentally realized current-based sensors can detect TATP either directly (using a polymer + Au nanoclusters substrate) [16] or via precursors (using a MoS2/reduced graphene oxide substrate) [17]. Other experimental methods utilize fluorescence [18] and cyclic voltammetry [20] with limited portability, and thermodynamic sensors [22]. Theoretical studies investigating TATP detection employ graphene/boron nitride [19] for detection via UV–vis absorption, and nitrogen-terminated carbon nanotubes for detection through changes in tunneling current [21]. Only a few of them provide contactless and continuous detection of TATP from the vapor phase [16, 22]. While some 2D materials, such as MoS_2_/RGO [17] or boron nitride flakes [19], were examined for the purpose of TATP detection, studies on the interaction of TATP and MXenes, and the potential for sensing are lacking. The MXene-based TATP resistive sensor could potentially offer greater portability, a larger signal-to-noise ratio, and low gas concentrations [23].

In this work, we numerically study electronic and transport properties of the Ti_3_C_2_O_2_ and Ti_3_C_2_F_2_ MXenes as potential TATP vapor sensing platforms using density functional theory (DFT) and non-equilibrium Green's functions (NEGF). We chose Ti_3_C_2_X_2_ (X = O, F) because surface termination may promote hydrogen bonding with TATP, conducting properties [24, 25], and a high signal-to-noise ratio [23]. Our results indicate that surface termination governs binding strength (hydrogen bonds) and charge-transfer direction. For Ti_3_C_2_F_2_, the TATP acts as an acceptor of negative charge, consequently increasing resistance (lowering electric current) of MXene upon adsorption. On the other hand, TATP donates a negative charge to Ti_3_C_2_O_2_, and has its highest occupied molecular orbital (HOMO) level pinned to the Fermi energy, thus producing a distinct transport fingerprint. These results advance the fundamental understanding of TATP adsorption and point towards novel sensor design.

## Calculation methods

**Fig. 1:**
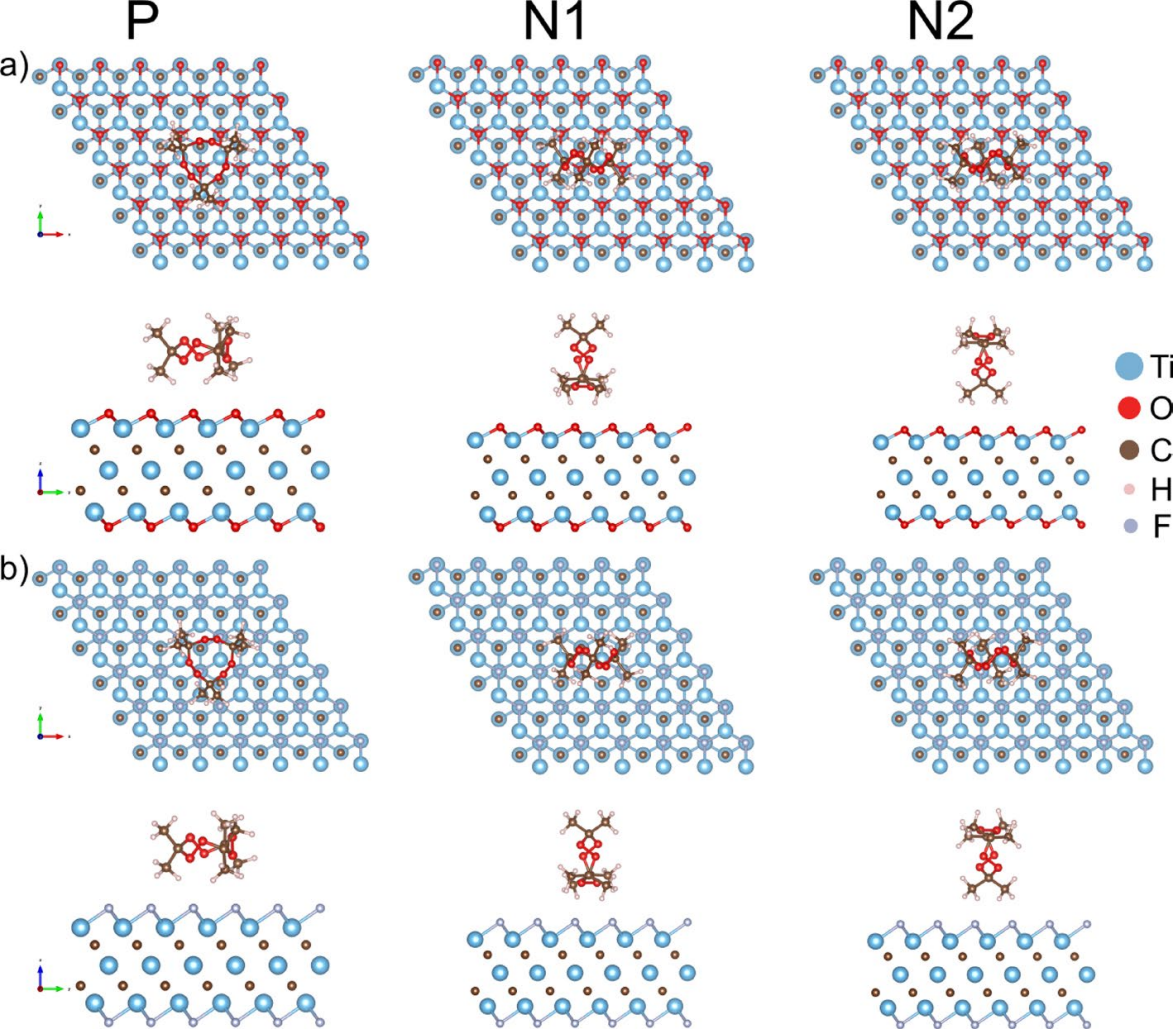
Top and side views of relaxed configurations of 6 × 6 **a** Ti_3_C_2_O_2_ and **b** Ti_3_C_2_F_2_ supercell with adsorbed TATP molecule in parallel (P) and two orthogonal configurations (orientations) with four (N1) and two (N2) TATP methyl groups close to the MXene surface

The calculations presented in this study were performed using the Siesta [26] (version 4.1.5) software package within generalized gradient approximation (GGA) with Perdew-Burke-Ernzerhof (PBE) [27] functional for the exchange–correlation term and the norm-conserving Troullier-Martins pseudopotentials [28]. We use the double-zeta polarized basis set for all calculations and the DFT-D2 Grimme correction [29] to account for the van der Waals interaction as implemented in Siesta. The unit cell parameters of the Ti_3_C_2_O_2_ and Ti_3_C_2_F_2_ were adopted from the paper by Khazaei et al., with the vacuum along the z direction being set at 40 Å to avoid the interaction between the repeated images [30]. The adsorption surfaces were formed by relaxing atomic positions while maintaining fixed unit cell constants. The post-relaxation band structures of the two MXenes, presented in Figure S1 of the Supporting Information, are in good agreement with previously reported results [25, 31]. We used 6 × 6 supercells of Ti_3_C_2_O_2_ and Ti_3_C_2_F_2_ (Fig. 1a and b, respectively). During the adsorption process, both the TATP molecule and the MXene supercell were subjected to full structural relaxation. For the geometry relaxation and calculation of the electronic properties of pristine and MXenes with the adsorbed TATP, the mesh-cutoff of 200 Ry and 9 × 9 × 1 k-point sampling were used. Siesta uses a localized basis set and introduces basis superposition error (BSSE). To account for BSSE, the counter-poise correction was applied to adsorption energies [32]. We calculate the adsorption energy according to the following formula:

$${E}_{ads}\left(SA\right)=\left(E\left(SA\right)-E\left({S}_{0}\right)-E\left({A}_{0}\right)\right)-{\delta }_{BSSE.}$$

The *E(SA)* is the total energy of the relaxed geometry of the given configuration (MXene slab and the TATP molecule), while *E(S*_*0*_*)* and *E(A*_*0*_*)* are those of the individually relaxed MXene slab and TATP, respectively. The *δ*_*BSSE*_ correction term is given by expression:

$${\delta }_{BSSE}={(E}_{SA}\left(A\right)-{E}_{SA}\left(S{A}_{G}\right))+{(E}_{SA}\left(S\right)-{E}_{SA}\left({S}_{G}A\right))$$

The *E*_*SA*_*(S)*/*E*_*SA*_*(A)* are the total energies of the MXene slab/molecule with coordinates from the relaxed geometry of the configuration SA. The *E(SA*_*G*_*)*/*E(S*_*G*_*A)* are total energies of the relaxed geometry of the configuration SA in which ghost orbitals are used to describe atomic species that belong to the molecule/MXene slab, respectively.

Transport calculations at finite bias were performed using TranSiesta and TBTrans (version 4.1.5) [33]. For the transport calculations, we use the 5 × 1x50 k-point sampling for the electrodes and 5 × 1x1 for the central region calculation, and all other parameters are the same as in Siesta calculations. The transmission curves and electric current at finite bias were obtained using TBTrans with 50 × 1x1 k-point sampling.

The current *I* through a molecule at finite bias *V* in TranSiesta follows the Landauer- Büttiker equation:

$$I\left(V\right)=2e/h \int\limits_{-\infty }^{+\infty }dE\left[f\left(E-{\mu }_{L}\right)-f\left(E-{\mu }_{R}\right)\right]T(E,V)$$

where *T*(*E*, *V*) is the transmission function spectrum, and *f*(*E*–*μ*_L/R_) is the Fermi–Dirac distribution in the left/right electrode with electrochemical potential *μ*_L/R_ = *E*_*F*_ ± eV/2.

The Siesta, TranSiesta, and TBTrans output data postprocessing was performed using sisl [34] and in-house-built scripts. The images of the geometries and charge density difference (CDD) plots were made using VESTA 3.5.8 software [35].

## Results and discussion

### TATP adsorption on MXene surface

To investigate whether Ti_3_C_2_O_2_ and Ti_3_C_2_F_2_ are viable for TATP sensing, we first calculate the adsorption energy. For both Ti_3_C_2_O_2_ and Ti_3_C_2_F_2_, we examine three different configurations (orientations) of TATP shown in Fig. 1 and designated as parallel (P) and orthogonal (N1 and N2). The calculated adsorption energies *E*_*ads*_, distances *d* (the shortest distance along the *z*-axis from the top oxygen/fluorine layer to the nearest H atom in the TATP molecule) and Hirshfeld charge excess at the TATP molecule *Q*_*TATP*_ for the three orientations are given in Table 1.

**Table 1:** The TATP adsorption energy (*E*_*ads*_), distance (*d*), and the Hirshfeld charge excess (*Q*_*TATP*_) at the molecule for three different orientations (see Fig. 1) at Ti_3_C_2_O_2_ and Ti_3_C_2_F_2_ surfaces

MXene	Configuration	*E*_*ads*_ (eV)	*d* (Å)	*Q*_*TATP*_ (e)
Ti_3_C_2_O_2_	P	− 0.45	2.0	+ 0.044
	N1	− 0.40	1.8	+ 0.050
	N2	− 0.28	2.0	+ 0.066
Ti_3_C_2_F_2_	P	− 0.37	1.8	− 0.163
	N1	− 0.36	1.7	− 0.153
	N2	− 0.10	1.8	− 0.141

Different numbers of TATP methyl groups are close to the surface for each orientation: three for P, four for N1, and two for N2. For each configuration (P, N1, N2), TATP adsorption energy is higher for Ti_3_C_2_O_2_ than for Ti_3_C_2_F_2_. For both MXenes, the adsorption energies of P and N1 are similar and significantly higher than *E*_*ads*_ for N2, since more methyl groups are available for interaction with the surface. The sign of *Q*_*TATP*_ is positive for Ti_3_C_2_O_2_ and negative for Ti_3_C_2_F_2_, meaning that TATP acts as an electron donor in the first and as an electron acceptor in the latter case. The absolute value of the *Q*_*TATP*_ is significantly higher for Ti_3_C_2_F_2_.

**Fig. 2:**
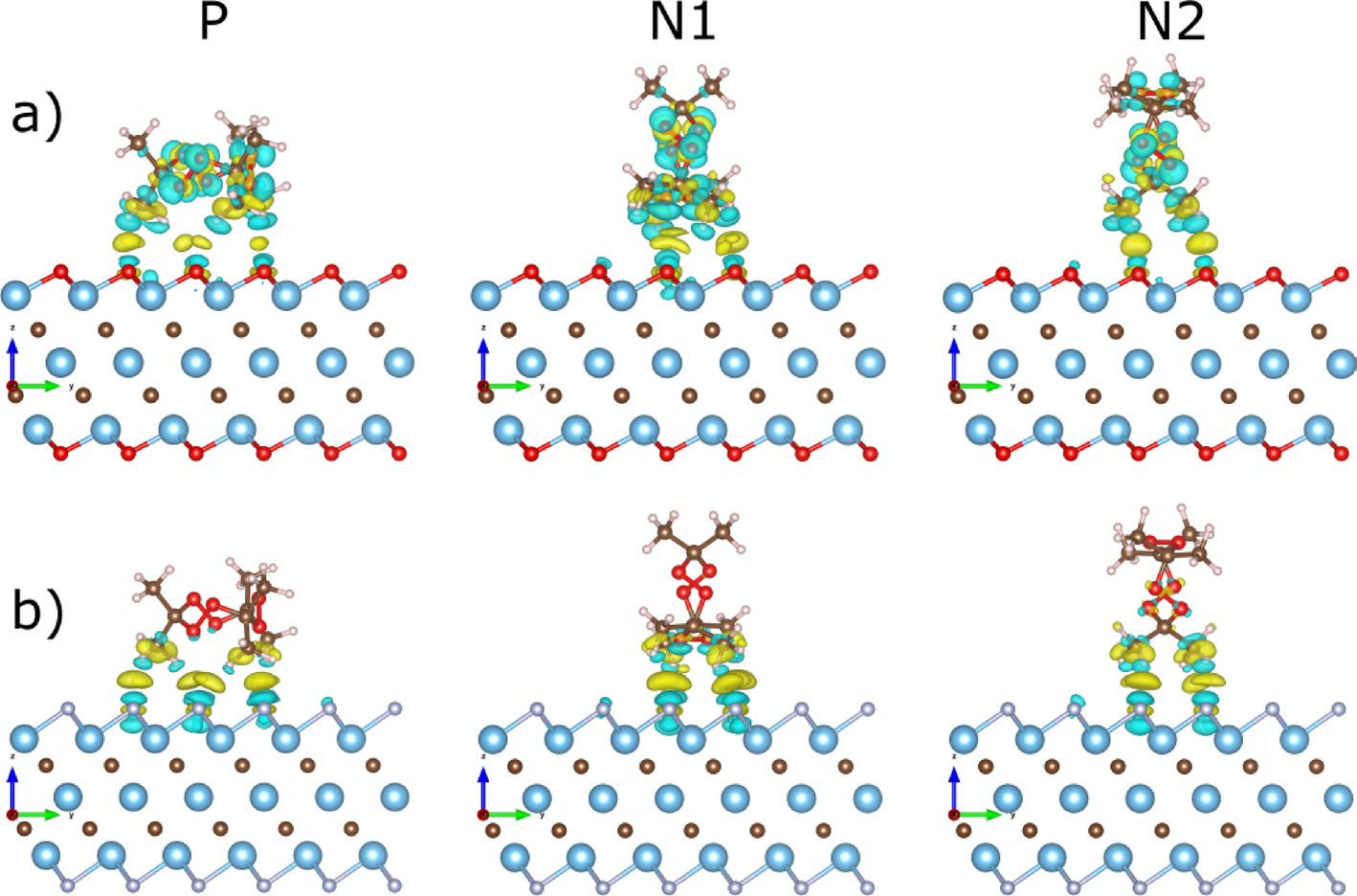
Charge density difference (CDD) plots for three different orientations (P, N1, and N2; see Fig. 1) of the TATP molecule adsorbed at a) Ti_3_C_2_O_2_ and b) Ti_3_C_2_F_2_ surface. Yellow/blue depict the electron accumulation/depletion, respectively. All CDD plots were obtained using the same isovalue of 0.005 e/Å^3^

To better understand the TATP adsorption on the surface of the MXenes, we plot the charge density difference (CDD) in Fig. 2. The CDD plots reveal significant differences of the adsorption for two terminations of MXenes in terms of charge redistribution. In the case of Ti_3_C_2_O_2_, the charge redistribution appears on the TATP oxygen ring (Fig. 2a), which is absent in the case of Ti_3_C_2_F_2_ (Fig. 2b). For all orientations and both MXenes we observe significant charge density differences in regions between TATP H atoms and the surface.

Depending on the orientation of a molecule, different numbers of hydrogen atoms (7, 5, and 4 for P, N1, and N2, respectively) of the TATP methyl groups interact with oxygen/fluorine atoms of MXene surfaces (Fig. 2a, b). To elucidate the nature of the interaction between TATP (P orientation) and the MXene surfaces, we performed a detailed chemical bonding analysis using Crystal Orbital Hamilton Populations (COHP) and bond lengths. Based on bond lengths from the Siesta output, the TATP molecule interacts with MXene surface via its hydrogen and carbon atoms. COHP analysis revealed that TATP molecule dominantly interacts with surface of MXenes via hydrogen implying hydrogen bonding mechanism. While hydrogen bonding represents the primary contribution to the total adsorption energy, bonding contributions from the TATP carbon atoms are negligible (~ − 10 ^−3^ eV) across both systems. For Ti_3_C_2_O_2_, we observed a minor orbital overlap between the surface Ti atoms and molecular H atoms, of the same order (~ − 10^−3^ eV).

The COHP curves for all relevant H-MXene termination interactions are provided in Figure S3 of the Supplementary Information. For both MXene systems, an extensive network (15 for Ti_3_C_2_F_2_ and 12 for Ti_3_C_2_O_2_) of hydrogen bonds with lengths ranging from 2.1 to 3.0 Å was identified; however, only a subset of these falls within the medium-to-strong interaction range (insets of Figure S3a, b), consistent with the CDD plots. The total integrated COHP (*ΣiCOHP*) is − 2 eV for Ti_3_C_2_O_2_ and − 3.09 eV for Ti_3_C_2_F_2_.

The *ΣiCOHP* value is significantly larger than *E*_*ads*_ for both MXenes, while the structural deformation is negligible for both the surface and molecule, implying that there is a significant contribution from Pauli repulsion [36]. The fact that Ti_3_C_2_F_2_ has lower *E*_*ads*_ implies stronger Pauli repulsion, which is consistent with closer proximity to the surface.

### Density of states

**Fig. 3:**
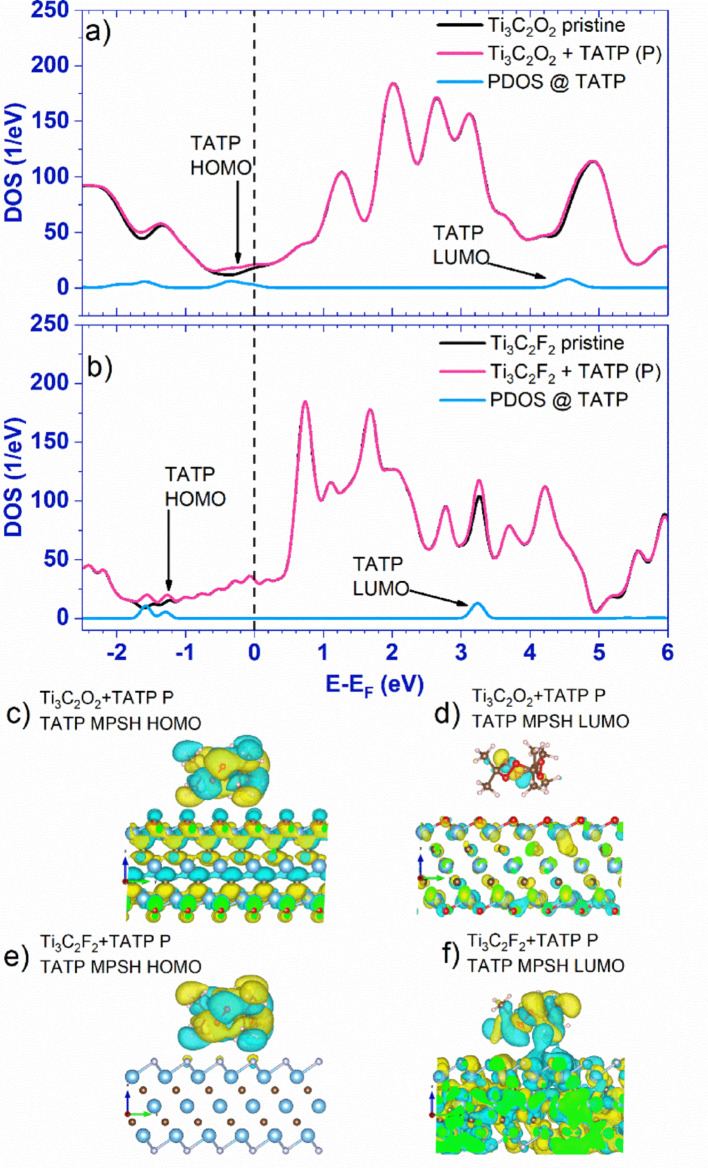
**a** The total density of states (DOS) for pristine (black) and Ti_3_C_2_O_2_ with an adsorbed TATP molecule (pink) and the DOS projected at the adsorbed TATP molecule (light blue). **b** The total DOS for pristine (black) and Ti_3_C_2_F_2_ with adsorbed TATP molecule (pink) and the DOS projected at the adsorbed TATP molecule (light blue). The Fermi energy is represented by a vertical dashed line, and the arrows indicate TATP HOMO and LUMO energies. Plot of TATP (Ti_3_C_2_O_2_ + TATP P)** c** HOMO and** d** LUMO orbitals, and (Ti_3_C_2_F_2_ + TATP P)** e** HOMO and **f** LUMO orbitals, with an isovalue of 0.01 e/Å^3^ used for all of the images

To gain insight into electronic interaction between the TATP molecule and two MXenes, we calculate the density of states (DOS) of pristine and MXenes with the adsorbed TATP molecule (0.2 eV smearing). Figure 3 shows the DOS analysis for pristine MXenes and MXene + TATP for the P configurations. The presence of adsorbed TATP molecule introduces changes in the DOS of both MXene + TATP systems (compare pink and black lines in Fig. 3a, b and Figure S2 of Supplementary information). The projected density of states (PDOS) at the TATP molecule (blue lines in Fig. 3a, b) confirms that these changes are caused by the TATP molecule since the total DOS of MXenes with adsorbed TATP differs from the pristine ones exactly at the PDOS TATP peak energies.

Density of states weakly depends on the orientation of the TATP molecule (Figure S2). In the case of Ti_3_C_2_O_2_, the broad TATP PDOS peak at − 0.3 eV, corresponding to TATP HOMO, HOMO-1, and HOMO-2 levels, lies close to the Fermi energy *E*_*F*_ (Fig. 3a) and, thus, the TATP HOMO is pinned to the Ti_3_C_2_O_2_ Fermi energy. In the combined MXene + TATP system, we assume the TATP HOMO as the first eigenstate below *E*_*F*_ characterized by a primary contribution from TATP atomic orbitals that correspond to the HOMO of the isolated molecule. We apply this same logic to define the LUMO, and utilize these terms consistently hereafter.

Hirschfeld charge excess *Q*_*TATP*_ (Table 1) is positive, indicating that negative charge was transferred from TATP to Ti_3_C_2_O_2_ and, according to the standard charge transfer theory [37], the alignment between two levels is achieved. Moreover, the CDD distribution (Fig. 2a) on the TATP molecule reflects the isolated TATP molecule HOMO wavefunction distribution, also pointing to Fermi level pinning [37].

There is no Fermi level pinning in the case for Ti_3_C_2_F_2_, since the *E*_*F*_ is close neither to the TATP HOMO (− 1.25 eV) nor LUMO (3.3 eV) (Fig. 3b). Standard charge transfer theory implies that no charge transfer should occur since neither HOMO nor LUMO are in proximity of *E*_*F*_ [37]. Nevertheless, according to Leenaerts et al. [38], charge transfer is still possible in such cases when there is a hybridization between a molecular orbital and the surface. The larger the spatial overlap between molecular and surface orbitals, and the smaller the energy difference between them stronger the hybridization [38].

The density of states analysis in Fig. 3b shows that there is a small energy difference between states in Ti_3_C_2_F_2_ and TATP HOMO and LUMO. That energy overlap is more pronounced for the LUMO state (3.3 eV) than for the HOMO (− 1.25 eV, Fig. 3b). In O-terminated MXene (Fig. 3a), the equivalent energy overlaps are not as pronounced as for the F-terminated one.

Next, we plot TATP HOMO and LUMO wavefunctions in Fig. 3c-f to explore the spatial overlap. For Ti_3_C_2_O_2_ (Fig. 3c, d), there is no significant spatial overlap between the HOMO or LUMO orbital and MXene. Therefore, there is no hybridization and thus no substantial charge transfer (Table 1).

For MXene with F termination (Fig. 3e, f), there is a significant spatial overlap only for the LUMO state. Notable energy and spatial overlaps imply hybridization between the LUMO and surface, and thus significant charge transfer (Table 1). That lack of hybridization in the case of the HOMO most likely stems from the absence of the spatial overlap, as the HOMO orbital is primarily located on the TATP oxygen ring.

### Electronic transport with adsorbed TATP

**Fig. 4:**
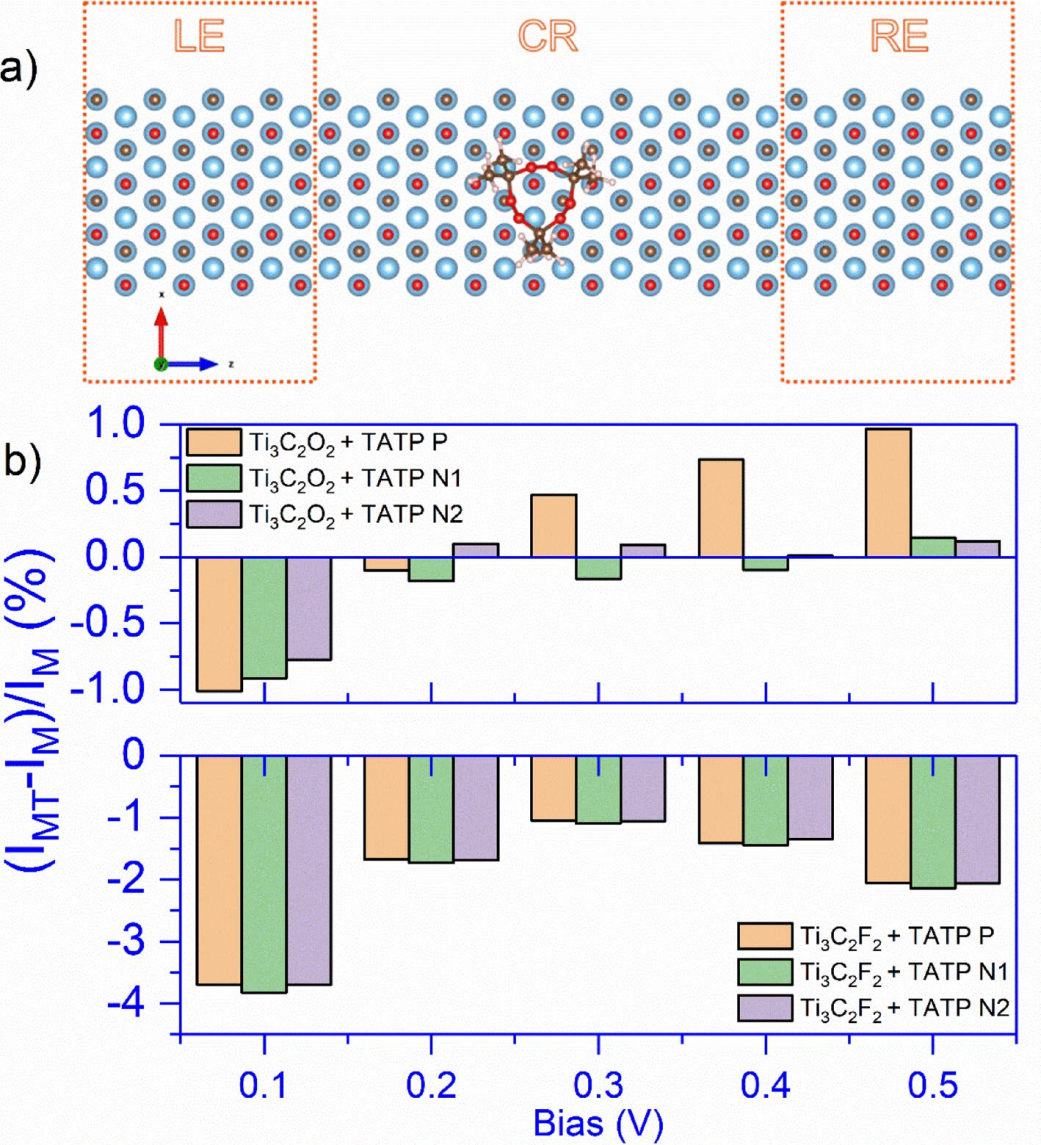
**a** The configuration used for the electronic transport calculations: the solid orange rectangles depict the left (LE) and right (RE) electrode regions, each consisting of four unit cells, with the eight-unit-cell central region (CR) in between. **b** Relative change of the electric current, 100% * (*I*_*MT*_* − I*_*M*_)/*I*_*M*_ (*I*_*M*_-current of pristine MXene and *I*_*MT*_-current of MXene + TATP), for studied bias values and TATP molecule orientations: Ti_3_C_2_O_2_ + TATP in top and Ti_3_C_2_F_2_ + TATP in bottom panel

To further examine the possibility of detecting TATP using the electric current (resistance), we have performed transport calculations for both MXenes without (pristine) and with adsorbed TATP molecule. Figure 4a shows the exemplary configuration used for the transport calculations of Ti_3_C_2_O_2_ with the adsorbed TATP molecule in the P orientation: left (LE) and right (RE) electrodes, consisting of four unit cells each, and the central region (CR) composed of eight unit cells. The transport direction is along *z-*axis, and the system is periodic along *x* and *y* directions; however, along *y* direction interaction of repeating images is avoided by choosing large cell constant of 40 Å.

Zero-bias transmission curves of MXenes, pristine and with adsorbed TATP, are given in Figure S4 (Supplementary Information). Upon TATP adsorption, only a small dip appears in transmission around − 0.06 eV, which coincides with TATP HOMO for Ti_3_C_2_O_2_ (Figure S4a), while for Ti_3_C_2_F_2,_ the transmission drops in the whole energy range (Figure S4b). The calculated *I–V* curves of both studied pristine MXenes are given in Figure S5 (Supplementary Information), and the obtained values, on the order of 10 μA, are in agreement with those previously reported [31, 39].

Figure 4b presents the relative change of current, 100% * (*I*_*MT*_–*I*_*M*_)/*I*_*M*_ (*I*_*M*_-current of pristine MXene and *I*_*MT*_-current of MXene + TATP) for both MXenes and different TATP orientations, and bias range 0.1–0.5 V.

For both MXenes, the highest relative change of current, 3.7% for Ti_3_C_2_F_2_ and 1% for Ti_3_C_2_O_2_, is observed for a bias value of 0.1 V, weakly dependent on molecule orientation. In the case of Ti_3_C_2_F_2_, the relative current change, first decreasing (for 0.2 and 0.3 V) and then slightly increasing (for 0.4 and 0.5 V), is independent of the molecule orientation. The electronic transmission for Ti_3_C_2_F_2_ is given in Figure S6: for all the bias values, the presence of the TATP molecule decreases the transmission and consequently the current. That larger current drop in the case of Ti_3_C_2_F_2_ is consistent with larger charge transfer and the fact that TATP acts as electron acceptor, which leads to an increase in MXene resistance[40].

**Fig. 5:**
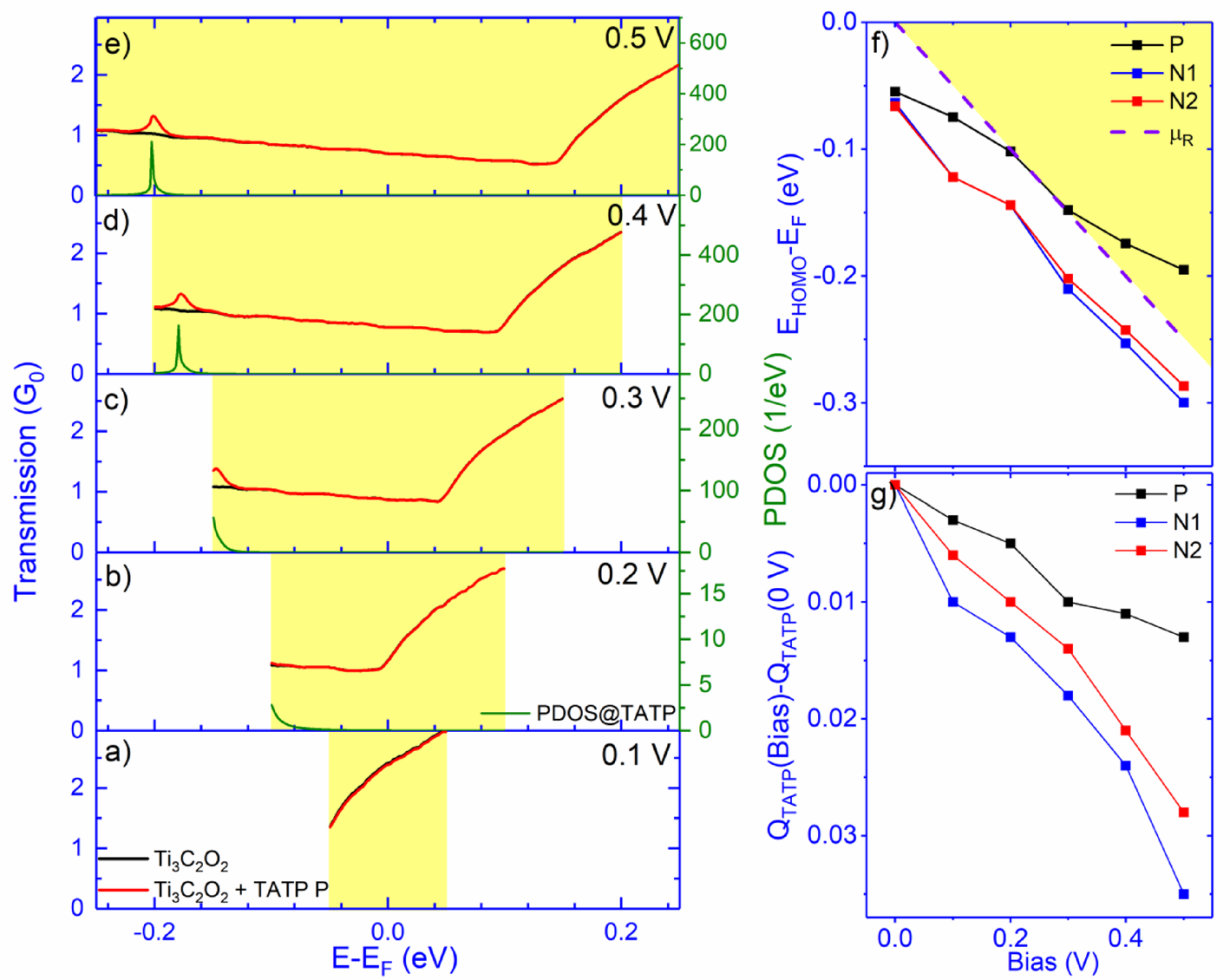
**a**–**e** Electronic transmission for pristine shown in bias window range (yellow rectangle), Ti_3_C_2_O_2_ (black line), and Ti_3_C_2_O_2_ + TATP P (red line) for bias values 0.1–0.5 V. **b**–**e** PDOS at TATP molecule (green line) for bias values. Bias voltage dependence of f) the TATP HOMO energy *E*_*HOMO*_ with respect to *E*_*F*_ and g) the change of charge *Q*_*TATP*_ – *Q*_*TATP*_(Bias = 0 V) located at the molecule for different molecular orientations (black, red, and blue correspond to P, N1, and N2 orientations, respectively). In **f**, the shaded region represents the bias window, whose boundary (dashed line) is defined by μ_R_—the chemical potential of the right electrode

In the case of Ti_3_C_2_O_2_, TATP’s orientation plays an important role for bias values higher than 0.1 V. While for N1 and N2 orientations, we observe negligible relative current changes, this for P, a steady relative current increase happens. To understand the origin of that current increase, we plot the electron transmission curves in the bias window range of pristine Ti_3_C_2_O_2_ (black curve) and Ti_3_C_2_O_2_ + TATP (pink curve) for bias values between 0.1 and 0.5 V (Fig. 5a–e). TATP adsorption induces a peak in transmission which originates from the TATP HOMO state: the peak enters the bias window at 0.3 V (Fig. 5c), causing the electric current to increase. The tail of the peak can be observed even at a bias voltage of 0.2 V (Fig. 5b); however, it does not significantly contribute to the current. To confirm that the peak originates from the TATP, we also plot the PDOS at the TATP molecule (Fig. 5b–e). It is clear that the PDOS@TATP peak coincides with the peak in the electronic transmission. Unlike for P, TATP HOMO for N1 and N2 orientations does not contribute to the electric current.

To show that, we plot the TATP HOMO energy *E*_*HOMO*_ with respect to *E*_*F*_ (Fig. 5f) and the change of Hirshfeld charge excess *Q*_*TATP*_(Bias)- *Q*_*TATP*_(Bias = 0 V) (Fig. 5g) located at the molecule for different bias values. While *Q*_*TATP*_ from transport calculations slightly differ from those from Table 1, the trend is the same: the P orientation has the lowest *Q*_*TATP*_ and is followed by N1 and N2 (0,035 e, 0,039 e, 0,061 e). Similar ordering is observed for *E*_*HOMO*_ with respect to *E*_*F*_, with *E*_*HOMO*_* – E*_*F*_ being smallest for P orientation. Upon the application of the bias, the positive charge located at the molecule increases, thus lowering the HOMO energy. In the case of P orientation, the change of charge and the HOMO energy are the slowest, and the HOMO peak gets “caught” by the bias window (the bias window boundary, i.e., the chemical potential changes faster than the HOMO energy with the bias). For N1 and N2, the rate of change of the charge is sufficient to “push” the HOMO energy away from the bias window, this is an example of strong pinning [41], resulting in the negligible resistance change (top panel in Fig. 4b).

Since Ti_3_C_2_X_2_ has an excellent signal-to-noise ratio [23], the relative electric current changes of 3.7 and 1.0% upon TATP adsorption should be measurable. Therefore, the Ti_3_C_2_O_2_ and Ti_3_C_2_F_2_ have the potential to be used as building blocks of a future TATP sensor based on electric transport, especially if used simultaneously as parts of the same device.

## Conclusions

This work provides a comprehensive first-principles analysis of TATP adsorption and detection on Ti_3_C_2_O_2_ and Ti_3_C_2_F_2_. The calculated adsorption energy values, together with bond length and COHP analysis, suggest that TATP is adsorbed at the surface of both MXenes through the formation of multiple hydrogen bonds. The analysis of DOS shows that TATP HOMO is pinned to the Fermi energy of Ti_3_C_2_O_2_, but not to the Ti_3_C_2_F_2_.

Further examination of electronic transport by NEGF theory shows that the TATP adsorption causes a decrease in the electric current (increases resistance) in Ti_3_C_2_F_2_ (all of the studied bias values) and Ti_3_C_2_O_2_ (0.1 and 0.2 V).

The largest relative current change, 3.7% and 1% for Ti_3_C_2_F_2_ and Ti_3_C_2_O_2_, respectively, was observed for 0.1 V. For Ti_3_C_2_O_2_ and bias values higher than 0.2 V, the electric current increases, consequently the relative current change also increases and reaches 0.95% at 0.5 V. Such behavior is a direct consequence of the e-transport mechanism, which is dominated by charge flow through the TATP’s molecular HOMO. This study establishes a basis for engineering MXene-based TATP sensors.

## Supplementary Information

Below is the link to the electronic supplementary material.

Supplementary Material 1

## Data Availability

The xyz coordinates of all relaxed configurations used in the computational work are deposited in a publicly accessible repository, Zenodo, 10.5281/zenodo.17722309.
