# Supplementary material for: First principles electronic transport study of triacetone triperoxide adsorbed on Ti3C2O2 and Ti3C2F2 MXene monolayers
Source: Discov Nano. 2026 Apr 21;21(1):133. doi: 10.1186/s11671-026-04561-2 (PMC13100224; doi:10.1186/s11671-026-04561-2)
Supplement: Supplementary file 1 — Supplementary Material 1 [file 11671_2026_4561_MOESM1_ESM.docx]

**Supplementary information**

**First principles electronic transport study of triacetone triperoxide adsorbed on Ti_3_C_2_O_2_ and Ti_3_C_2_F_2_ MXene monolayers**

Aleksandar Ž. Tomović,^a,*^, Ivana Djurišić, ^a^ Miloš S. Dražić, ^a^ Vladimir P. Jovanović ^a^ and Radomir Zikic^a^

^a^ University of Belgrade - Institute for Multidisciplinary Research, National Institute of the Republic of Serbia, Kneza Višeslava 1, Belgrade, 11030, Serbia

* Corresponding Author: Aleksandar Ž. Tomović. E-mail: *aleksandar.tomovic@imsi.bg.ac.rs*







**Figure S1** Band structure of unit cell of Ti_3_C_2_F_2_ and Ti_3_C_2_O_2_ after relaxation of atomic positions.





**Figure S2** a) The density of states (DOS) for pristine Ti_3_C_2_O_2_ (black) and with adsorbed TATP molecule in P, N1, and N2 configurations (solid pink, red, and blue lines, respectively) and the projected DOS (dashed pink, red, and blue lines, respectively) at the TATP molecule. b) The density of states (DOS) for pristine Ti_3_C_2_F_2_ (black) and with adsorbed TATP molecule in P, N1, and N2 configurations (solid pink, red, and blue lines, respectively) and the projected DOS (dashed pink, red, and blue lines, respectively) at the TATP molecule.





**Figure S3** COHP curves of interactions between H atoms of the TATP molecule in P orientation and a) F atoms of Ti_3_C_2_F_2_ and b) O atoms of Ti_3_C_2_O_2_ surface. Insets show bond length versus integrated value of COHP (iCOHP).





**Figure S4** Zero-bias transmission of different TATP orientations for a) pristine Ti_3_C_2_O_2_ and with adsorbed TATP in P, N1 and N2 orientations (black, red, blue, green curves, respectively), b) Ti_3_C_2_F_2_ and with adsorbed TATP in P, N1 and N2 orientations (black, red, blue, green curves, respectively).





**Figure S5** The calculated *I-V* curves of pristine Ti_3_C_2_O_2_ (black) and Ti_3_C_2_F_2_ (red).





**Figure S6** a-e) Electronic transmission for pristine shown in bias window range (yellow rectangle), Ti_3_C_2_F_2_ (black line), and Ti_3_C_2_F_2_ + TATP P, N1, and N2 (red, blue, and green line, respectively).
